# IFN-γ-response mediator GBP-1 represses human cell proliferation by inhibiting the Hippo signaling transcription factor TEAD

**DOI:** 10.1042/BCJ20180123

**Published:** 2018-09-25

**Authors:** Bea Unterer, Veit Wiesmann, Mekala Gunasekaran, Heinrich Sticht, Clara Tenkerian, Jürgen Behrens, Marina Leone, Felix B. Engel, Nathalie Britzen-Laurent, Elisabeth Naschberger, Thomas Wittenberg, Michael Stürzl

**Affiliations:** 1 Division of Molecular and Experimental Surgery, Translational Research Center, Department of Surgery, University Medical Center Erlangen, Friedrich-Alexander-Universität Erlangen-Nürnberg (FAU), Schwabachanlage 12, 91054 Erlangen, Germany; 2 Fraunhofer Institute for Integrated Circuits IIS, Department of Image Processing and Medical Engineering, Am Wolfsmantel 33, 91058 Erlangen, Germany; 3 Chair of Experimental Medicine II, Nikolaus-Fiebiger-Zentrum, Friedrich-Alexander-Universität Erlangen-Nürnberg (FAU), Glückstrasse 6, 91054 Erlangen, Germany; 4 Division of Bioinformatics, Institute of Biochemistry Friedrich-Alexander-Universität Erlangen-Nürnberg (FAU), Fahrstraße 17, 91054 Erlangen, Germany; 5 Experimental Renal and Cardiovascular Research, Translational Research Center, Department of Nephropathology, Institute of Pathology, University Medical Center Erlangen, Friedrich-Alexander-Universität Erlangen-Nürnberg (FAU), Schwabachanlage 12, 91054 Erlangen, Germany

**Keywords:** cell proliferation, colorectal cancer, guanylate-binding proteins, interferons

## Abstract

Interferon-gamma (IFN-γ) is a pleiotropic cytokine that exerts important functions in inflammation, infectious diseases, and cancer. The large GTPase human guanylate-binding protein 1 (GBP-1) is among the most strongly IFN-γ-induced cellular proteins. Previously, it has been shown that GBP-1 mediates manifold cellular responses to IFN-γ including the inhibition of proliferation, spreading, migration, and invasion and through this exerts anti-tumorigenic activity. However, the mechanisms of GBP-1 anti-tumorigenic activities remain poorly understood. Here, we elucidated the molecular mechanism of the human GBP-1-mediated suppression of proliferation by demonstrating for the first time a cross-talk between the anti-tumorigenic IFN-γ and Hippo pathways. The α9-helix of GBP-1 was found to be sufficient to inhibit proliferation. Protein-binding and molecular modeling studies revealed that the α9-helix binds to the DNA-binding domain of the Hippo signaling transcription factor TEA domain protein (TEAD) mediated by the ^376^VDHLFQK^382^ sequence at the N-terminus of the GBP-1-α9-helix. Mutation of this sequence resulted in abrogation of both TEAD interaction and suppression of proliferation. Further on, the interaction caused inhibition of TEAD transcriptional activity associated with the down-regulation of TEAD-target genes. In agreement with these results, IFN-γ treatment of the cells also impaired TEAD activity, and this effect was abrogated by siRNA-mediated inhibition of GBP-1 expression. Altogether, this demonstrated that the α9-helix is the proliferation inhibitory domain of GBP-1, which acts independent of the GTPase activity through the inhibition of the Hippo transcription factor TEAD in mediating the anti-proliferative cell response to IFN-γ.

## Introduction

IFN-γ regulates many cellular processes including innate and acquired immune responses, host defense reactions, and exerts direct anti-tumorigenic effects [1,2]. The large GTPase human guanylate-binding protein 1 (GBP-1) is highly induced by IFN-γ and indicates activation of cells by this cytokine [3,4] in diseased tissues in inflammatory bowel diseases, colorectal carcinoma (CRC), and others [5–9]. In CRC, GBP-1 acts as a tumor suppressor and its expression is correlated with a prolonged 5-year cancer-related survival [8,10]. Functional analyses showed that GBP-1 is a cellular mediator of the anti-tumorigenic activities of IFN-γ. It mediates the inhibition of proliferation, spreading, migration, and invasion of both epithelial tumor cells and blood vessel endothelial cells, and reduces tumorigenicity of CRC tumor cells in experimental animal models [5,11–13]. The structure of GBP-1 is divided into a large N-terminal globular (Glo) GTPase domain and a purely α-helical (Hel) domain containing the α-helices 7–13 and a C-terminal CAAX isoprenylation motif that regulates the membrane association of the protein [14,15]. At present, it is not known which mechanism is involved in the inhibition of cell proliferation.

In cancer research, the Hippo signaling pathway has attracted an increasing amount of attention as alterations have been detected in various cancers [16–19]. The Hippo pathway is regulated by the Yes-associated protein (YAP) and its paralogue transcriptional co-activator with PDZ-binding motif (TAZ) [17]. Depending on the phosphorylation status, YAP/TAZ can translocate into the nucleus, where they bind to the transcription factor TEA domain protein (TEAD) and activate the expression of genes that promote cell proliferation [17]. TEAD is involved in cancer by regulating the expression of cancer-associated [20] or prognostic genes [21,22]. Furthermore, inhibition of TEAD suppressed proliferation and oncogenic transformation in mouse embryos and mouse NIH3T3 and human HEK293 cells [17]. As yet, the interaction of anti-proliferative IFN-γ and cell growth-activating Hippo signaling has not been investigated.

Here, we investigated on the specific mechanisms responsible for the anti-proliferative effect of GBP-1. Our findings connect the anti-proliferative IFN-γ effects with the Hippo signaling pathway and showed that the α9-helix of GBP-1 is sufficient for this effect but does not modify other cellular responses to GBP-1. This may open new avenues for dissecting the complex cellular response to IFN-γ.

## Materials and methods

### Cells, plasmids, and transfections

DLD1 (CCL-221) and HeLa (CCL-2) cells were purchased from ATCC. Cells were authenticated by the German Collection of Microorganisms and Cell Cultures (DSMZ) using nonaplex PCR DNA profiling of eight highly polymorphic sites of short tandem repeats [23]. DLD1 cells were cultured in RPMI 1640 (Gibco, Thermo Fischer Scientific) and HeLa cells in DMEM (Gibco, Thermo Fischer Scientific), both supplemented with 2 mM glutamine (PAA) and 10% (v/v) FBS (PAA). Cells were maintained at 37°C with 5% (DLD1) or 8.5% (HeLa) CO_2_ and 95% humidity. Cells were routinely tested for the absence of mycoplasma contamination using the MycoAlert mycoplasma detection kit (Lonza).

The plasmids pMCV2.2(−) containing a gentamycin resistance cassette and pMCV1.4(−) were obtained from Mologen. The expression cassette provided a Flag-tag sequence (F) in the 5′ direction of the cloning site. CDNA encoding full-length GBP-1 or fragments thereof were inserted into these plasmids in frame with the Flag-tag. GBP-1 alanine mutants (A1-7/α) were generated using the GeneArt® Site-Directed Mutagenesis PLUS Kit (Thermo Fischer Scientific) according to the manufacturer's protocol. The following subfragments of GBP-1 (NM_002053.2) were generated: globular domain (amino acid residues 1–290), helical domain (291–592), α7–11 (311–478), α7–9 (311–424), α9–11 (376–478), α12–13 (484–582), α9 (376–424), and α7–13 (311–582).

DLD1 cells were transfected with the pMCV2.2 plasmids (stable cell lines) or pMCV1.4 plasmids (transient transfections) using PolyFect (Qiagen) according to the manufacturer's protocol. For the generation of stable DLD1 cell lines, transfected cells were selected by the addition of G418 (500 µg/ml, PAA). HeLa cells were transfected using the calcium phosphate method [24]. Endogenous GBP-1 expression in HeLa cells was induced by treatment with human recombinant IFN-γ (Roche) in the indicated concentrations. For all experiments, cells were harvested at 80% confluence.

### 5-Ethynyl-2′-deoxyuridine incorporation assay and automatic image analysis

**Figure 1.**
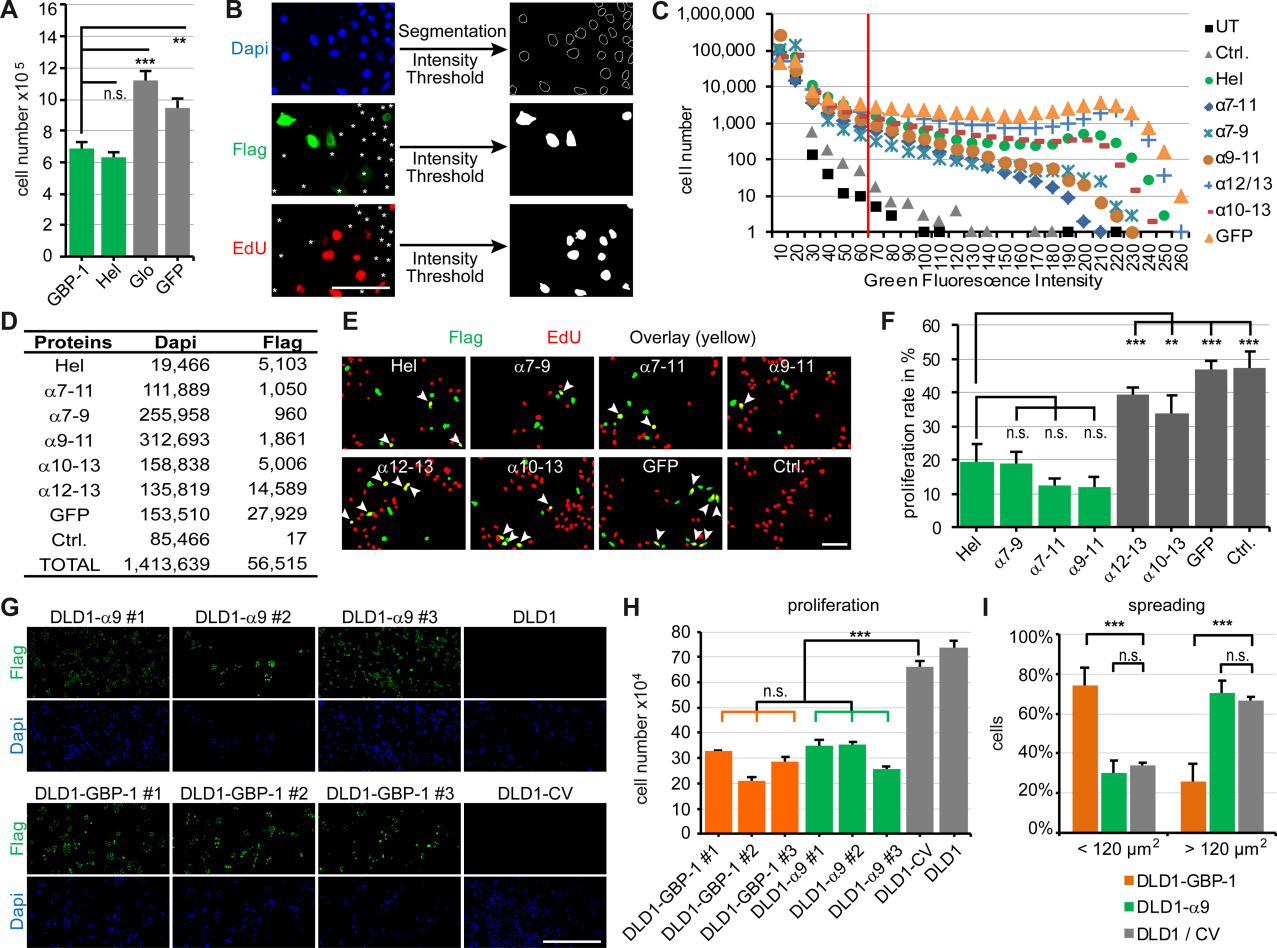
The α9-helix of GBP-1 is sufficient to inhibit cell proliferation of colorectal carcinoma cells. (**A–H**) DLD1 cells were transiently transfected with the indicated Flag-tagged expression plasmids, the plain vector (Ctrl.) or were left untransfected (UT). (**A**) Proliferation was determined by cell counting after 4 days (*n* = 3). (**B–H**) Proliferation of transfected cells was analyzed using EdU. Scale bar represents 80 µm. (**B**) Detection of total cells (DAPI, blue), transfected cells (Flag, green) and proliferating cells (EdU, red). Representatives of nuclei segmentation (top, right) and signal thresholds (middle, lower right) as applied in the automatic signal detection procedure are shown for cells expressing α12–13. Cells exhibiting fluorescence intensity below the threshold are indicated by asterisks (left). (**C**) Distribution of green fluorescence intensities. A fluorescence threshold (red vertical line) was chosen that discriminates between non-transfected and transfected cells (above the threshold) and included high numbers of transfected cells for the subsequent quantitative evaluation of the proliferation rates. (**D**) Numbers of cells analyzed (total (Dapi) and transfected (Flag)) are given. (**E**) Representative results of the automatic cell detection (transfected cells (green), EdU-positive cells (red), transfected proliferating cells (yellow, arrows)). (**F**) Proliferation rates of exclusively transfected cells are shown for all conditions except for Ctrl., where the proliferation rate of all cells was calculated (*n* = 3). Green bars indicate transfections where the α9-helix was present. (**G**) Flag-tagged GBP-1-α9 (DLD1-α9 #1, #2, #3) or GBP-1 full-length protein (DLD1-GBP-1 #1, #2, #3) expressed in three independent single-cell-derived lines were detected by Flag staining (green). Cell nuclei are stained with Dapi. Cells stably transfected with the control vector (DLD1–CV) or untransfected cells (DLD1) were used as negative controls. Scale bar represents 500 µm. (**H**) Proliferation of stably transfected DLD1 cells was determined by cell counting after 7 days (*n* = 3). (**I**) Spreading of transfected cell was analyzed by determining the cell surface area 20 min after seeding. Percentages of cells possessing an area below 120 µm^2^ and above 120 µm^2^ are shown (*n* = 6). Control cells (DLD1/CV) included DLD1–CV and untransfected DLD1 cells.

For these analyses, 1.5 × 10^4^ DLD1 cells per chamber were seeded in 1.5% gelatin-coated four-chamber culture slides (Falcon, BD Biosciences) and were transiently transfected 24 h later. Forty-eight hours after transfection, 5-ethynyl-2′-deoxyuridine (EdU, 10 µM) was added to the cells for 3 h. Cells with active DNA synthesis were detected using the Click-iT^™^ Plus EdU Alexa Fluor^™^ 555 Imaging Kit (Thermo Fisher Scientific, C10638) according to the manufacturer's protocol. Ectopically expressed proteins were detected using a rabbit-α-Flag antibody (Abcam, catalog ABR-01098, 1 : 500) and AlexaFluor488-conjugated goat-α-rabbit IgG secondary antibody (Thermo Fisher Scientific, A11034, 1 : 500). The slides were mounted using Roti-Mount Fluor Care Dapi (Roth). Images were acquired in an automatic process using an AxioScanZ1 (Zeiss). DAPI images were preprocessed with a difference of Gaussian (DoG) filter consisting of a first Gaussian filter with lower sigma to remove background artifacts and a second Gaussian filter with a higher sigma to remove image noise. The regions showing nuclei were delineated from the background by adaptive thresholding with a *k*-means clustering algorithm on the preprocessed image [25]. The source image and the result image of the thresholding step were subjected to the hybrid watershed algorithm to delineate single possible nuclei regions [26]. As a post-processing step, the average intensity of these regions was calculated, and only regions showing a minimum average intensity *I*_DAPI_ were regarded as nuclei (Figure 1B). The Flag images were preprocessed identically using the resulting image of the thresholding step and the positions of the previously segmented nuclei from the Dapi channel as input. For the nuclear EdU marker, the positions of the nuclei regions were used. Identified regions were post-processed with the minimum intensity threshold as presented for the nuclei. Proliferation rates represent the ratio between transfected proliferating cells and total transfected cells in all conditions except in control-transfected cells, where the ratio of total proliferating cells to total cells was calculated.

### Cell counting proliferation assay

Transiently transfected DLD1 cells were seeded in triplicates for each condition (3 × 10^4^ cells/well) in six-well plates and transfected 24 h later. Four days after transfection, total cell numbers per well were determined using the Coulter-Counter Z2 (Beckman). Each stably transfected single-cell-derived line was seeded in triplicates (3 × 10^3^ cells/well) in 24-well plates, and total cell numbers were determined 7 days later. In both cases, the medium was renewed every 2 days.

### Spreading assay

The assay was performed in fibronectin-coated 96-well plates as previously described [13] using 8 × 10^3^ DLD1 cells per well. Surface areas of the cells were measured 20 min after seeding using Image J. The experiment was performed once using triplicates of two DLD1-GBP-1 clones, two DLD1-α9 clones, the DLD1–CV clone and untransfected DLD1 cells. Cells possessing an area above and below 120 µm^2^ were classified.

### Co-immunoprecipitation and western blot analysis

Transfected DLD1 or HeLa cells were used for co-immunoprecipitations as describe previously [27] using 20 µl Flag M2 monoclonal antibody affinity gel (Sigma–Aldrich, A2220). For the precipitation of endogenous GBP-1, beads were prepared by covalent cross-linking of the α-GBP-1 antibody (1B1) [7] as described before [28].

Precipitated proteins and cell lysates were separated under reducing conditions in 10–15% sodium dodecyl sulfate–polyacrylamide gels and transferred onto a Hybond-P polyvinylidene difluoride membrane (GE Healthcare). Membranes were blocked for 1 h with 5% skim milk in 1× PBS and incubated overnight (4°C) with the following primary antibodies: mouse-α-Flag M2 (Sigma, F3165, 1 : 10 000), rabbit-α-β-actin (Abcam, ab8227, 1 : 5000), rabbit-α-panTEAD (Abcam, ab197589, 1 : 1000), rabbit-α-TEAD1 (Abcam, ab133535, 1 : 1000), rabbit-α-TEAD2 (Avia Systems Biology, OAAB18572, 1 : 1000) or rabbit-α-TEAD3 (Santa Cruz, L-17, sc-102130, 1 : 500), and rat-α-GBP-1 (1B1 [7,28], hybridoma supernatant, 1 : 500). The respective secondary antibodies (Dako, 1 : 5000) were coupled to horseradish peroxidase (incubation for 1 h). Signals were detected with the ECL Prime Western Blotting System and the Amersham Imager 600 (both GE Healthcare). Quantification was performed using the ImageQuantTL software (GE Healthcare).

### Luciferase activity assay

DLD1, DLD1–CV or DLD1-GBP1 (#1–3) cells were seeded in triplicates in a 12-well plate. DLD1 cells were transfected 24 h later with a mixture of TEAD-dependent luciferase reporter and pRenilla (Promega) using PolyFect. HeLa cells were seeded in a 12-well plate in duplicates for each condition. RNA silencing was performed by reverse transfection using Lipofectamine™ RNAiMAX in OptiMEM medium (both Thermo Fischer Scientific) when indicated. The following small interfering RNAs (siRNA) were used: GBP-1 Stealth Select RNAi™ siRNA (HSS104020) and Stealth RNAi™ Negative Control Duplexes (Medium GC) (Thermo Fischer Scientific) at a final concentration of 8 nM. The medium was renewed 24 h later, and 10 U/ml IFN-γ was added or not as indicated, followed by transfection of the TEAD-dependent luciferase reporter, a YAP expression plasmid (pEGFP-YAP, Addgene) [29] and pRenilla (Promega) using the calcium phosphate method. The DLD1 and HeLa cells reached 70% confluency 30 h after transfection. Cells were lysed, and luciferase activity was measured using a dual-luciferase reporter assay system (#E1960; Promega) following the manufacturer's instructions. The transfection efficiency was normalized to the thymidine kinase promoter-driven Renilla luciferase activity as an internal control.

### RNA isolation and RT-qPCR

RNA was isolated using the RNeasy Mini Kit (Qiagen). Subsequently, DNA was removed by RNase-free DNase (Ambion) treatment, and RNA yield was determined using a Nanodrop 2000c (PeqLab, VWR). In all cases, the standard protocols of the manufacturer were followed.

RT-qPCR primers and 5′-FAM-3′-TAMRA-labeled probes were designed and checked for specificity using PrimerBlast (NCBI). RT-qPCR (TaqMan) was performed as described recently [10]. Briefly, the absence of residual DNA was confirmed by DNA-specific primers for the *progestagen-associated endometrial protein (PAEP)* gene. All samples were normalized using RPL37A (mean of triplicates) as a reference gene. Subsequently, the ΔΔCT method (ΔCt_Ctrl. _− ΔCt_GBP-1_) was used for the calculation of the respective fold changes ($2^{-\Delta \Delta C_{t}}$) for each target gene. Measurements were performed two times, each in triplicates.

The primers and probes were purchased from Eurogentec. The DNA primer/probe sequences were as follows: *paep* (gene ID 5047): forward CACAGAATGGACGCCATGAC, probe AGCCCTCAGCCCTGCTCTCCATC, reverse AAACCAGAGAGGCCACCCTAA; the RNA primer/probe sequences were as follows: *rpl37a* (NM_000998.4): forward TGTGGTTCCTGCATGAAGACA, probe TGGCTGGCGGTGCCTGGA, reverse GTGACAGCGGAAGTGGTATTGTAC; *ctgf* (GI 98986335): forward AGCAGACTCAGCTCTGACATT, probe TGTTCAGGAATCGGAATCCTGTCGA, reverse AGGCAAATTCACTTGCCACA; *c-myc* (GI 1059791704): forward AACCAGGTAAGCACCGAAGT, probe TGAGTCGAATGCCTAAATAGGGTGTCT, reverse CAATAGCGCAGGAATGGGAGA; *focm1* (GI 340545539): forward GAGCTGAAGGGTGGGAACAA, probe TGCCCAGCAGTCTCTTACCTTCCC, reverse GGACCACCCTGCAAAGATCA.

### Yeast two-hybrid screening

Yeast two-hybrid (Y2H) assays were performed in the yeast L-40 strain. The sequence of GBP-1-α9 (residues 376–424) was cloned into the bait vector pBTM116. This bait was then screened against a mouse embryonic day E9.5–E10.5 library in pVP16 as described previously [30]. A total of 71 clones were picked and tested for growth in medium without histidine. The strong positive candidates were selected for DNA isolation and analyzed by sequencing.

### Computer-assisted molecular docking

Docking was performed with the program GRAMM-X [31] using the known structures of GBP-1 (PDB: 1DG3) [15] and of the DNA-binding domain of TEAD1 (PDB: 2HZD) [32] as the input. Based on the experimental data, GBP-1-α9 (residues 376–424) was defined as the interaction site, whereas no restraints were made on the binding mode of TEAD1. The 10 top scoring docking solutions were further analyzed and visualized using RasMol [33].

### Statistical analyses

Data are presented as the mean ± SD. Statistical differences were calculated by the unpaired two-tailed *t*-test (luciferase, qPCR, and immunoprecipitation) or ANOVA (SPSS) for multiple groups followed by a *post hoc* analysis (Tukey with Levene's test *P* > 0.05). *P*-values <0.05 were considered statistically significant (*P < 0.05, **P < 0.01, ***P < 0.001). All studies were conducted in triplicates and repeated at least three times independently if not stated otherwise.

## Results

### The α9-helix of GBP-1 is sufficient to inhibit cell proliferation

Previously, the helical domain (Hel) of GBP-1 was shown to inhibit proliferation of endothelial cells, while the globular domain (Glo) was unnecessary for this effect [5]. To translate this finding to colorectal cancer cells, we used DLD1 cells as a model system because they lack endogenous GBP-1 expression [1]. A transient transfection approach confirmed that the isolated helical domain inhibits proliferation of DLD1 cells (Figure 1A).

To determine which of the seven α-helices forming the helical domain were responsible for the suppression of proliferation, Flag epitope-tagged GBP-1 fragments with different combinations of α-helices were expressed in DLD1 cells. The impact of the GBP-1 fragments on proliferation was examined at the single-cell level using computer-assisted image analyses of EdU staining (proliferation marker, red) in cells expressing the recombinant proteins (green) (Figure 1B–E). Cell proliferation rates (double-positive/green) were significantly reduced in cells expressing protein fragments containing the helix α9 (α7–9, α7–11 and α9–11) (Figure 1F, green). The inhibition obtained with these parts was comparable to the inhibition observed with the complete helical domain (Figure 1F). In contrast, in cells expressing other parts of the helical domain (α12–13 or α10–13) or GFP, proliferation was not inhibited and was similar to that of the empty vector-transfected cells (Figure 1F, gray).

To investigate whether the isolated α9-helix of GBP-1 is sufficient for the suppression of proliferation, the α9-helix (DLD1-α9) and full-length GBP-1 (DLD1-GBP-1) were stably expressed in DLD1 cells. To exclude clonal effects, three independent single-cell-derived lines were generated. GBP-1 and GBP-1-α9-helix expression were confirmed using immunocytochemical staining (Figure 1G). Cells stably transfected with the empty control vector (DLD1–CV) and non-transfected DLD1 cells were used as negative controls (Figure 1G). All cell lines expressing GBP-1 or the GBP-1-α9-helix exhibited a significantly decreased cell proliferation in comparison with DLD1–CV and non-transfected DLD1 cells (Figure 1H). This demonstrated that the 49 amino acids of the GBP-1-α9-helix are sufficient to inhibit cell proliferation.

Previously, we reported that GBP-1 inhibits spreading of endothelial cells in a GTPase-dependent manner [13]. In accordance with this, cell spreading was significantly reduced in DLD1-GBP-1 cells in comparison with the spreading of control cells (Figure 1I). In contrast, cell spreading was not affected in DLD1-α9 cells, which showed a similar spreading pattern as the control cells (Figure 1I). Moreover, we investigated whether the α9-helix is able to remodel the actin cytoskeleton as it was previously reported for wild-type GBP-1 [28]. Transient expression of Flag epitope-tagged GBP-1 in HeLa cells resulted in the complete reorganization of the actin cytoskeleton and the disruption of stress fibers compared with control-transfected cells (Supplementary Figure S1). On the contrary, expression of Flag epitope-tagged α9-helix did not perturb the actin cytoskeleton. These observations demonstrated that the α9-helix selectively inhibits proliferation and has no impact on other cellular activities regulated by GBP-1 such as cell spreading and actin remodeling.

### The α9-helix of GBP-1 interacts with the transcription factor TEAD

To investigate the mechanism by which the GBP-1-α9-helix may inhibit cell proliferation, we searched for cellular-binding proteins using a Y2H screen [34]. The α9-helix of GBP-1 was used as bait and a well-established mouse E9.5–E10.5 cDNA library as prey [30,35,36]. Out of all putative interacting molecules, TEAD was the most promising candidate because TEAD has been shown to activate cell proliferation, and five independent but partly overlapping clones were identified. Specifically, these clones shared the residues 53–141 (corresponding to human TEAD2 isoform 3), which are part of the DNA-binding domain of TEAD (Supplementary Figure S2A).

**Figure 2.**
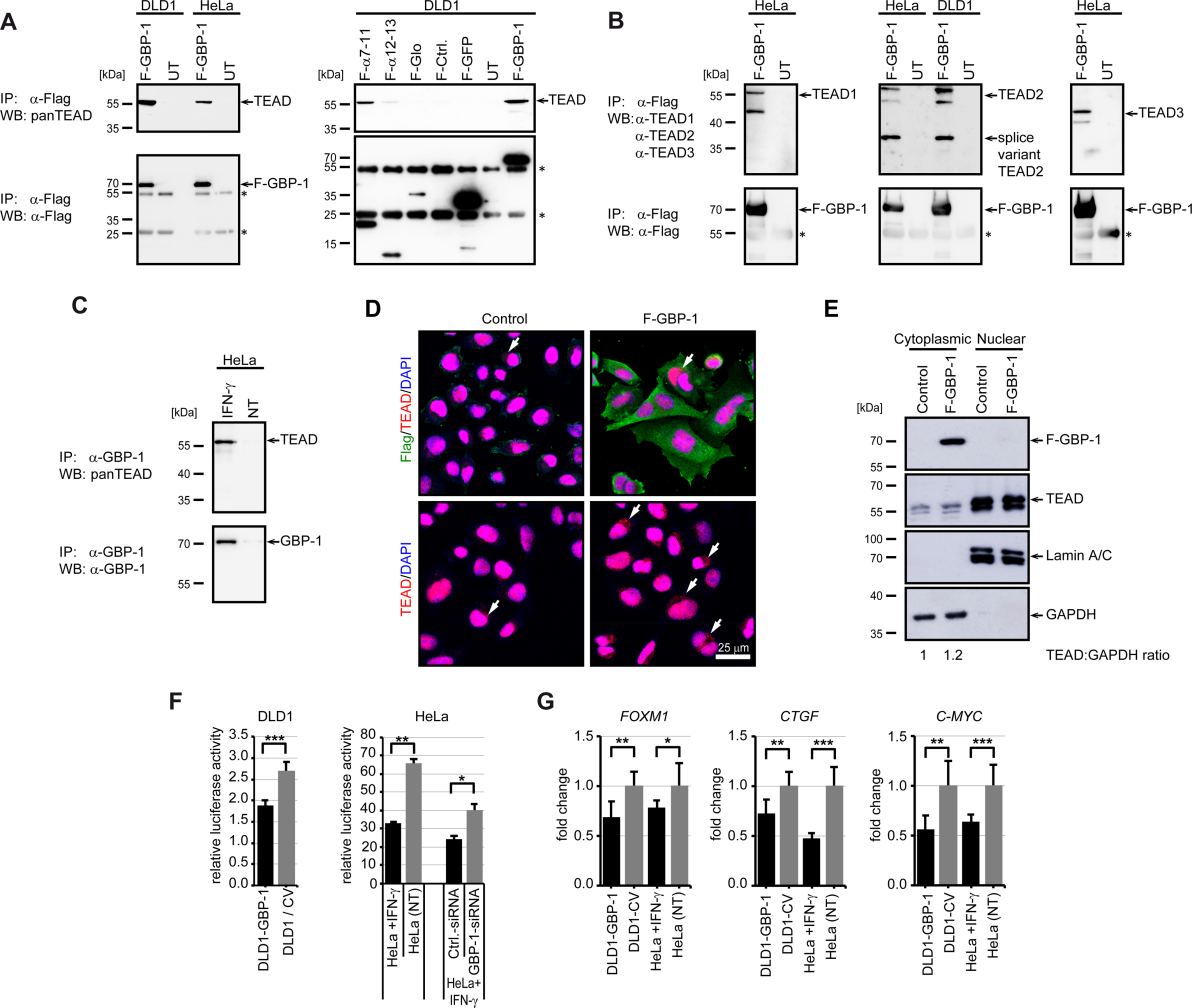
GBP-1 interacts with and inhibits TEAD. (**A** and **B**) DLD1 or HeLa cells were either left untransfected (UT) or transiently transfected with Flag-tagged GBP-1 (F-GBP-1), respective fragments (F-α7–11, F-α12–13, F-Glo), GFP (F-GFP) or Ctrl. and subjected to co-immunoprecipitation (top). Expression of transfected Flag-tagged proteins and their binding to the beads were detected by Flag immunoprecipitation and subsequent western blot (bottom). Immunoglobulin heavy and light chains are indicated by asterisks. All proteins showed a clear signal at the corresponding sizes. (**A**) After α-Flag co-precipitation, TEAD was detected by western blot using a pan-antibody. (**B**) After α-Flag co-precipitation, TEAD1, TEAD2 and TEAD3 were detected by western blot using specific antibodies (upper). (**C**) HeLa cells were either stimulated with 50 U/ml IFN-γ or were not treated (NT) and subjected to co-immunoprecipitation. After co-precipitation, TEAD was detected by western blot using a pan-antibody (top). Expression of endogenous GBP-1 and its binding to the beads was detected by α-GBP-1 immunoprecipitation and subsequent western blot (bottom). No immunoglobulin chains appeared as the antibody was covalently linked to the beads. (**D**) Immunofluorescent double staining of F-GBP-1 (green, anti-Flag) and TEAD (red, anti-pan TEAD) in DLD1 cells stably transfected by either control vector (control) or human GBP-1 (F-GBP-1). Nuclei were counterstained by DAPI (blue). Staining was recorded by a confocal microscope. Size bar indicates 25 µm. Cytoplasmic localization of TEAD is indicated by white arrows. (**E**) Cells from (**D**) were fractionated and analyzed by western blotting using anti-Flag and anti-pan TEAD antibodies. Lamin A/C and GAPDH were used as controls for the nuclear and cytoplasmic fractions, respectively. The ratio of TEAD relative to GAPDH was quantified using ImageJ and normalized to the control cells. (**F**) Activity of a TEAD-dependent luciferase reporter was measured in DLD1-GBP-1 (*n* = 6) cells and compared with the control cells (DLD1/CV) showing the mean of six experiments with control vector-transfected (*n* = 3) or untransfected DLD1 cells (*n* = 3). In HeLa cells (right), the luciferase activity was determined in IFN-γ-treated (10 U/ml) and non-treated (NT) cells (*n* = 2). SiRNA against GBP-1 (GBP-1-siRNA) and control siRNA (Ctrl.-siRNA) were used as indicated. Data shown were normalized to Renilla luciferase activity. (**G**) mRNA expression of FOXM1 (left), CTGF (middle) and C-MYC (right) were measured by TaqMan qPCR in GBP-1 or control vector (CV)-stably transfected DLD1 cells and IFN-γ- (100 U/ml) treated or non-treated (NT) HeLa cells (*n* = 6).

In the validating co-immunoprecipitation experiments, endogenous TEAD showed a very strong interaction with ectopically expressed Flag-tagged GBP-1 in DLD1 and HeLa cells (Figure 2A, left). Furthermore, TEAD also interacted with the GBP-1-α7–11 fragment, which included the α9-helix (Figure 2A, right). TEAD was not precipitated using the α12–13 helices, the globular domain, or negative controls such as the empty vector (Ctrl.), GFP, or lysates of untransfected cells (Figure 2A, right). These results showed that ectopically expressed GBP-1 interacts with endogenous TEAD in mammalian cells.

Next, the interaction with the specific TEAD family members TEAD1–3 was investigated. An interaction of ectopic GBP-1 with endogenous TEAD1, TEAD2 and TEAD3 was demonstrated (Figure 2B) and an interaction of endogenous GBP-1 with endogenous TEAD was shown in IFN-γ-stimulated HeLa cells (Figure 2C). Moreover, slightly increased amounts of cytoplasmic TEAD were detected in DLD1 cells stably expressing Flag epitope-tagged GBP-1, both in immunocytochemical staining (Figure 2D, arrows) and in Western blot analyses of cytoplasmic and nuclear cell fractions (Figure 2E).

To investigate the functional impact of the GBP-1–TEAD interaction, we investigated TEAD-associated transcriptional activation in mammalian cells in the absence and presence of GBP-1 using a TEAD-dependent luciferase reporter assay (Figure 2F). In stably transfected DLD1 cells, TEAD transcriptional activity was significantly lower in the presence of GBP-1 (DLD1-GBP-1) compared with its activity in control cells (Figure 2F, left). Furthermore, when GBP-1 expression was induced with IFN-γ in HeLa cells, TEAD activity was also repressed (Figure 2F, right). This IFN-γ-associated decrease in TEAD activity was reverted when GBP-1 expression was inhibited by a specific siRNA (Figure 2F, right). Western blot analysis of the luciferase lysates demonstrated that the siRNA against GBP-1 inhibited GBP-1 expression (Supplementary Figure S2B). In a next step, quantitative RT-PCR was used to investigate whether the expression of the TEAD-target genes, *foxm1* [37,38], *c-myc* [39], and *connective tissue growth factor* (*ctgf*) [40], were influenced by GBP-1. The integrity of the isolated RNA and primer specificity are shown in Supplementary Figure S2C,D. Each of the different TEAD-target genes was down-regulated in the presence of GBP-1 regardless of whether GBP-1 was ectopically expressed or induced by IFN-γ (Figure 2G). These results indicate that GBP-1 inhibits the transcriptional activity of the Hippo transcription factor TEAD.

### GBP-1 inhibits proliferation by binding to TEAD

To analyze whether TEAD binding is responsible for the inhibition of proliferation, we aimed to determine the TEAD-binding site in the GBP-1 sequence and to investigate whether mutation of this site abrogates the anti-proliferative effect of GBP-1.

**Figure 3.**
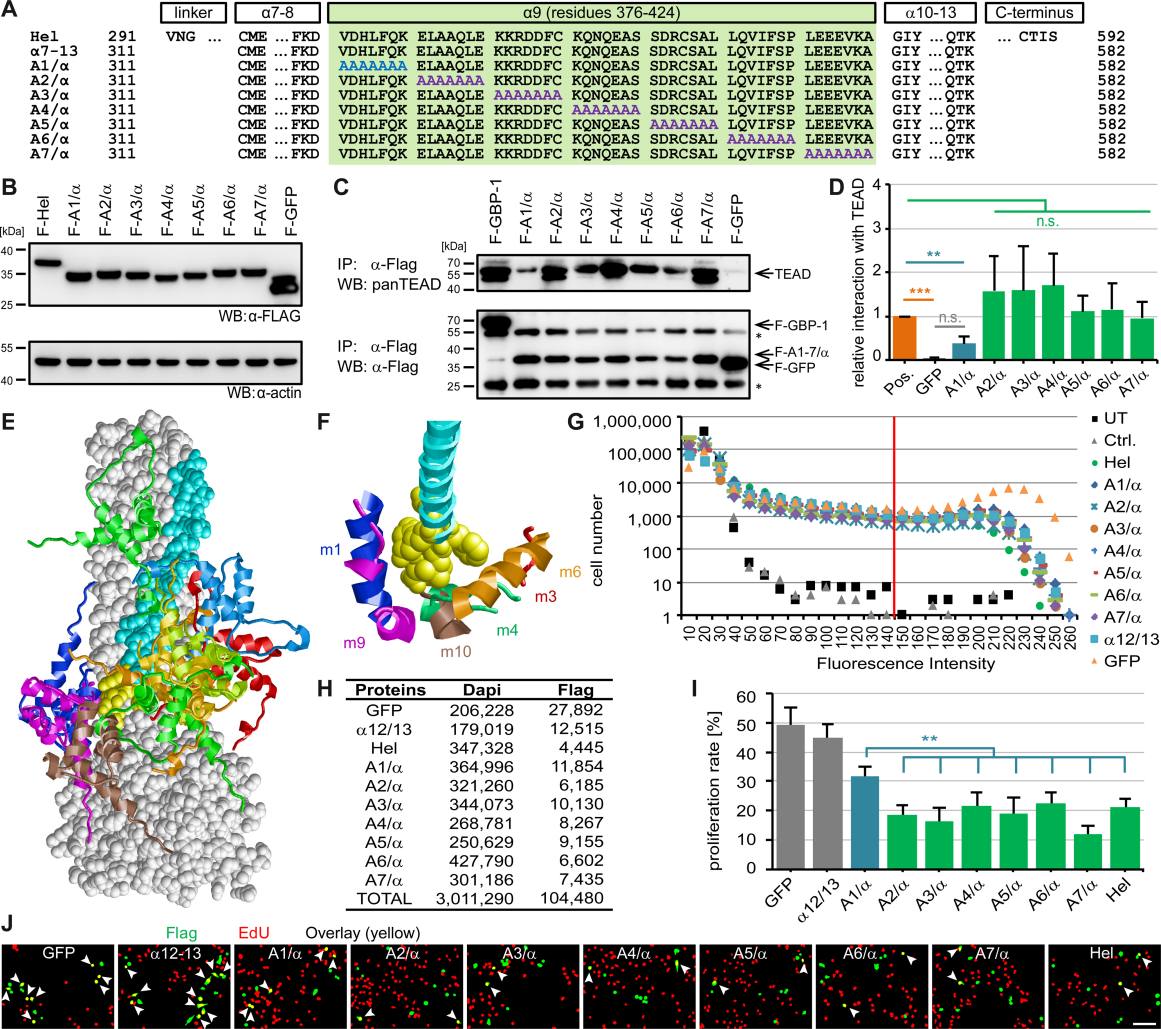
GBP-1 inhibits proliferation by interacting with TEAD. (**A**) Amino acid sequences of indicated GBP-1 fragments and mutants are shown. Alanine substitutions within the α9-helix (green) are highlighted in blue/purple. The A1-7/α mutants begin at the α7 and end after the α13 lacking the CAAX motif and contain an N-terminal Flag tag. (**B**) Indicated proteins were detected in whole-cell lysates of transiently transfected HeLa cells via the Flag epitope. (**C**) Lysates shown in (**B)** were subjected to α-Flag co-precipitation. TEAD was detected by western blot using a pan-antibody (top). Expression of transfected Flag-tagged proteins and their binding to the beads was detected by Flag immunoprecipitation and subsequent western blot (lower). (**D**) Quantification of three independent co-immunoprecipitation experiments (*n* = 3). TEAD signal was divided by Flag-per-IgG signal followed by normalization to positive control (pos.) (pos. shows the mean of three experiments with GBP-1 (*n* = 2) or wild-type α7–13 (*n* = 1)). (**E** and **F**) Mode of TEAD GBP-1 interaction derived from docking simulations. GBP-1 is shown in space-filled presentation (gray) with residues 376–382 highlighted in yellow and the remaining parts of the α9-helix in cyan. The TEAD1 docking models are shown in ribbon presentation and colored individually per model. (**E**) Distribution of the 10 top scoring TEAD1 docking models (m1–m10). Residues 27–105 containing the DNA-binding domain are shown. (**F**) Detailed view of TEAD1 atoms that are closer than 10 Å to the GBP-1 residues 376–382 are shown in ribbon presentation (m1, blue; m3, red; m4, green; m6, orange; m9, magenta; m10, brown). The view is rotated by ∼70° around the horizontal axis compared with e. (**G–J**) Proliferation of transiently transfected DLD1 cells was analyzed using EdU (*n* = 9). (**G**) Distribution of green fluorescence intensities and applied threshold (red line). (**H**) Numbers of cells analyzed (total (Dapi), transfected (Flag)). (**I**) Proliferation rates represent the ratio between transfected proliferating and total transfected cells. Experiment was performed independently three times, each in triplicates. (**J**) Representative results of the automatic cell detection (transfected cells (green), EdU-positive cells (red), and transfected proliferating cells (yellow, arrows)). Scale bar represents 80 µm.

The best candidate site within GBP-1 was the α9-helix, as this helix was used in the original Y2H screen and GBP-1 fragments lacking the α9-helix failed to interact with human TEAD in co-immunoprecipitation experiments. To narrow down the specific TEAD-binding site in the GBP-1-α9-helix, a mutational screen was performed. Flag-tagged mutant variants (A1–7/α) harboring consecutive cassettes of seven alanines to substitute all of the 49 amino acids of the α9-helix were generated (Figure 3A). All of the A1-7/α mutants begin at the α7 and end after the α13 lacking the CAAX motif and contain an N-terminal Flag tag. The mutants were expressed after transient transfection in HeLa cells (Figure 3B). TEAD showed a significantly reduced interaction with the A1/α mutant (residues 376–382) compared with its interaction with GBP-1 and all other mutants (A2-7/α) (Figure 3C,D). The use of an anti-pan TEAD antibody resulted in multiple bands in those lanes where high amounts of TEAD were precipitated, which likely represent different TEAD molecules. The amount of TEAD co-precipitated with the A1/α mutant was comparable with the TEAD amount precipitated with the negative control protein GFP (Figure 3C,D). To further validate the relevance of the seven N-terminal residues of the α9-helix as TEAD-binding site, a computer-assisted molecular docking analysis was performed. The entire GBP-1-α9 helix and the available structure of the DNA-binding domain of TEAD1 were defined as docking partners without imposing any additional constraints on the geometry of the interaction. The DNA-binding domain was chosen as it was the only motif consistently present in each of the five interaction fragments identified in the Y2H analysis (Supplementary Figure S2A,E). Analysis of the 10 most favorable docking solutions revealed that TEAD binding was not evenly distributed along the α9-helix. Eight of the 10 solutions clustered around the N-terminal part of the α9-helix, indicating the existence of a preferred docking site (Figure 3E). In six of the binding models, the distance between TEAD residues and the GBP-1 amino acid sequence ^376^VDHLFQK^382^, which was targeted by the A1/α mutant, was below 10 Å (Figure 3F). Accordingly, in agreement with the experimental data, the molecular docking analysis demonstrated that the N-terminal sequence of GBP-1-α9 was the preferred interaction site within TEAD. Of note, the ^376^VDHLFQK^382^ sequence was not detected in other human proteins, with the exception of GBP-3 (Supplementary Table S1), which is the closest homolog of GBP-1 [4].

Next, the effects of the A1-7/α mutants on proliferation were determined in transiently transfected DLD1 cells using Flag (transfected cells in green) and EdU (proliferating cells in red) staining (Figure 3G–J). More than 3 million cells were subjected to single-cell image analysis (Figure 3H). Cells expressing the A1/α mutant exhibited a significantly higher proliferation rate than those expressing one of the other mutants (A2-7/α) or the helical domain (Figure 3I). Representative results are shown in Figure 3J. Both the A1/α and A2/α mutant induced remodeling of the actin cytoskeleton, supporting that the anti-proliferative effect was not due to the impact of GBP-1 on the cytoskeleton (Supplementary Figure S1).

These results demonstrated that the first seven residues of the α9 helix caused the anti-proliferative effect of GBP-1 by binding to and functionally inhibiting the Hippo transcription factor TEAD.

## Discussion

Dissection of the manifold activities of GBP-1 at the structure–function level is required to improve the understanding of the cellular functions of IFN-γ-induced large GTPases. Here, we elucidated the molecular mechanism of the GBP-1-mediated suppression of proliferation. We showed that the α9-helix of GBP-1 is sufficient to inhibit cell proliferation, regulated by the binding of a seven-amino acid motif (^376^VDHLFQK^382^) of the N-terminus of the GBP-1-α9-helix to the Hippo transcription factor TEAD. Mutation of the ^376^VDHLFQK^382^ sequence leads to abrogation of both the anti-proliferative activity of GBP-1 and the TEAD binding. This binding lead to a functional deactivation of TEAD as confirmed concordantly with different experimental approaches. Of note, the inhibition of cell spreading by GBP-1, which requires the GTPase function located in the globular domain of the protein, was not affected by the α9-helix. Altogether, this demonstrates that the α9-helix is a specific proliferation inhibitory domain of GBP-1, which acts by binding to TEAD via the ^376^VDHLFQK^382^ sequence, leading to the functional neutralization of the transcription factor. A recent report showing that overexpression of TEAD1 increases colorectal cancer cell proliferation while its knockdown reduces it clearly supports our findings [41]. Furthermore, the interaction with GBP-1 could be confirmed for TEAD1–3.

Expression analyses showed that three well-established TEAD-target genes (*foxm1*, *ctgf*, and *c-myc*) were down-regulated in cells expressing GBP-1. FOXM1 is a transcription factor that promotes cell cycle progression [42]. CTGF has been described to be down-regulated in the presence of IFN-γ [43]. The transcription factor C-MYC is encoded by a proto-oncogene and is involved in the development of many different kinds of tumors [44]. Accordingly, the down-regulation of these genes is well in agreement with the anti-proliferative and anti-tumorigenic function of GBP-1. The results were confirmed in DLD1 cells that lack endogenous GBP-1 [11] and HeLa cells that exhibit a functional IFN-γ response, demonstrating that the impact of GBP-1 on TEAD does not require additional IFN-γ-induced factors.

The binding of GBP-1 to TEAD was confirmed with several different approaches: first, a Y2H screen, second, immunoprecipitation, and third, a computer-assisted molecular docking analysis. For the latter, the DNA-binding domain of TEAD1 was used for the following reasons: (i) an experimental structure was available representing the conformation in the absence of DNA as required for the modeling of cytoplasmic interactions [32], (ii) the TEAD1 domain exhibits a high sequence identity with and covers most parts of the TEAD2 interaction fragments identified in the Y2H analysis (Supplementary Figure S2A,E), and (iii) the interaction of GBP-1 with TEAD1 was experimentally confirmed. The mode of interaction differed between the different docking solutions. This is frequently observed for unbiased docking simulations in the absence of experimental restraints. Interestingly, the analysis of the 10 most favorable docking solutions revealed that 80% of the solutions clustered around the ^376^VDHLFQK^382^ sequence. This strongly supported experimental data, identifying the ^376^VDHLFQK^382^ sequence as a TEAD interaction motif.

In a previous study, GBP-1 was reported to inhibit proliferation by decreasing levels of β-catenin, the executioner molecule of the Wnt pathway [45]. Notably, TEAD activation by YAP/TAZ leads to activation of serum/glucocorticoid-regulated kinase 1 (SGK1), which is a known inhibitor of glycogen synthase kinase 3β (GSK3β), the central component of the β-catenin destabilization complex [46,47]. Accordingly, TEAD inhibition by GBP-1 may result in increased GSK3β expression, subsequent β-catenin degradation and finally inhibition of cell proliferation. In agreement with this hypothesis, a slight increase in β-catenin activity was observed in GBP-1-expressing cells in the presence of a GSK3β inhibitor (supplementary figure 6 of ref. [36]).

Our study demonstrates that the 49 amino acids of the α9-helix are both necessary and sufficient for the inhibition of cell proliferation. Accordingly, this fragment or even a sub-fragment such as the ^376^VDHLFQK^382^ sequence may be used to generate therapeutic peptides, for instance, to induce the deactivation of TEAD within anti-tumorigenic strategies. Support for the applicability of such an approach has been provided in a previous report [48]. *Vice versa*, peptides interfering with the GBP-1–TEAD interaction could allow cell proliferation under inflammatory conditions. This second approach may be of specific relevance to support cell growth and angiogenesis in non-healing wounds.

Interestingly, many other binding partners of GBP-1 have been identified previously. This includes proteins encoded by different viruses associated with anti-viral effects as well as cellular-binding factors, which binding triggers distinct processes of the IFN-γ response (Table 1). In some cases, the GTPase activity is required, whereas in others, it's dispensable, indicating that different areas of the protein and different mechanisms were involved. These observations support in combination with our results of the back tracing of a specific non-GTPase-dependent GBP-1 function to a defined area of the protein the modular structure of the multifunctional GBP-1 and opens new avenues to exploit specific activities of IFN-γ for targeted clinical applications.

**Table 1:** Published GBP-1-binding partners and GBP-1 functions

	Binding^1^	Function
Virus-encoded proteins		
HCV-NSB5 protein	GTPase activity required [49]	Anti-viral [49]
IAV-NS1 protein	GTPase activity required [50]	Anti-viral [50]
KSHV RTA	n.d.	Anti-viral [51]
Cell-encoded proteins		
Actin	n.d.[28]^2^	Anti-viral [51]^2^
PIM1	Globular and helical domain, partly overlapping with TEAD-binding motif [52]	Improved cell survival, resistance to chemotherapy [52]
Plastin-2 [53]	n.d.	n.d.
PRL-3	n.d.	May suppress PRL-3-mediated p53 activity [54]
Spectrin β-chain	n.d.	Inhibition of T-cell receptor signaling [53]
TEAD	α9-helix of the helical domain^3^	Inhibition of cell proliferation^3^

Abbreviations: **HCV**, Hepatitis C virus; **IAV**, Influenza A virus; **KSHV**, Kaposi's sarcoma-associated herpesvirus; **n.d.**, exact binding region and/or impact of binding on function has not been investigated; **NS1**, nonstructural protein 1; **NSB5**, nonstructural protein B5; **PIM1**, proviral integration of Moloney virus 1; **PRL**, phosphatase of regenerating liver-3; **RTA**, replication and transcription activator; **TEAD**, TEA domain protein.

1 Specific binding area or dependence of binding on GTPase activity is given.

2 Own unpublished results suggest that binding occurs in the helical domain, and GTPase activity may modulate binding, inhibiting cell migration and spreading.

3 The results of the present work.

## Abbreviations

CRC: colorectal carcinoma
*ctgf*: *connective tissue growth factor*
EdU: 5-ethynyl-2′-deoxyuridine
GBP-1: guanylate-binding protein 1
GSK3β: glycogen synthase kinase 3β
IFN-γ: interferon-gamma
TEAD: TEA domain protein
Y2H: yeast two-hybrid
YAP: Yes-associated protein

## References

[1] Britzen-Laurent N., Herrmann C., Naschberger E., Croner R.S. and Stürzl M. (2016) Pathophysiological role of guanylate-binding proteins in gastrointestinal diseases. World J. Gastroenterol. 22, 6434–6443 10.3748/wjg.v22.i28.6434 27605879 PMC4968125

[2] López-Posadas R., Stürzl M., Atreya I., Neurath M.F. and Britzen-Laurent N. (2017) Interplay of GTPases and cytoskeleton in cellular barrier defects during gut inflammation. Front. Immunol. 8, 1240 10.3389/fimmu.2017.01240 29051760 PMC5633683

[3] Cheng Y.S., Colonno R.J. and Yin F.H. (1983) Interferon induction of fibroblast proteins with guanylate binding activity. J. Biol. Chem. 258, 7746–7750 PMID:6305951

[4] Tripal P., Bauer M., Naschberger E., Mortinger T., Hohenadl C., Cornali E. et al. (2007) Unique features of different members of the human guanylate-binding protein family. J. Interferon Cytokine Res. 27, 44–52 10.1089/jir.2007.0086 17266443

[5] Guenzi E., Töpolt K., Cornali E., Lubeseder-Martellato C., Jörg A., Matzen K. et al. (2001) The helical domain of GBP-1 mediates the inhibition of endothelial cell proliferation by inflammatory cytokines. EMBO J. 20, 5568–5577 10.1093/emboj/20.20.5568 11598000 PMC125279

[6] Haep L., Britzen-Laurent N., Weber T.G., Naschberger E., Schaefer A., Kremmer E. et al. (2015) Interferon γ counteracts the angiogenic switch and induces vascular permeability in dextran sulfate sodium colitis in mice. Inflamm. Bowel Dis. 21, 2360–2371 10.1097/MIB.0000000000000490 26164664

[7] Lubeseder-Martellato C., Guenzi E., Jörg A., Töpolt K., Naschberger E., Kremmer E. et al. (2002) Guanylate-binding protein-1 expression is selectively induced by inflammatory cytokines and is an activation marker of endothelial cells during inflammatory diseases. Am. J. Pathol. 161, 1749–1759 10.1016/S0002-9440(10)64452-5 12414522 PMC1850787

[8] Naschberger E., Croner R.S., Merkel S., Dimmler A., Tripal P., Amann K.U. et al. (2008) Angiostatic immune reaction in colorectal carcinoma: impact on survival and perspectives for antiangiogenic therapy. Int. J. Cancer 123, 2120–2129 10.1002/ijc.23764 18697200

[9] Naschberger E., Wenzel J., Kretz C.C., Herrmann M., Stürzl M. and Kuhn A. (2011) Increased expression of guanylate binding protein-1 in lesional skin of patients with cutaneous lupus erythematosus. Exp. Dermatol. 20, 102–106 10.1111/j.1600-0625.2010.01160.x 21121962

[10] Naschberger E., Liebl A., Schellerer V.S., Schutz M., Britzen-Laurent N., Kolbel P. et al. (2016) Matricellular protein SPARCL1 regulates tumor microenvironment-dependent endothelial cell heterogeneity in colorectal carcinoma. J. Clin. Invest. 126, 4187–4204 10.1172/JCI78260 27721236 PMC5096916

[11] Britzen-Laurent N., Lipnik K., Ocker M., Naschberger E., Schellerer V.S., Croner R.S. et al. (2013) GBP-1 acts as a tumor suppressor in colorectal cancer cells. Carcinogenesis 34, 153–162 10.1093/carcin/bgs310 23042300

[12] Guenzi E., Töpolt K., Lubeseder-Martellato C., Jörg A., Naschberger E., Benelli R. et al. (2003) The guanylate binding protein-1 GTPase controls the invasive and angiogenic capability of endothelial cells through inhibition of MMP-1 expression. EMBO J. 22, 3772–3782 10.1093/emboj/cdg382 12881412 PMC169055

[13] Weinlander K., Naschberger E., Lehmann M.H., Tripal P., Paster W., Stockinger H. et al. (2008) Guanylate binding protein-1 inhibits spreading and migration of endothelial cells through induction of integrin alpha4 expression. FASEB J. 22, 4168–4178 10.1096/fj.08-107524 18697840

[14] Praefcke G.J. and McMahon H.T. (2004) The dynamin superfamily: universal membrane tubulation and fission molecules? Nat. Rev. Mol. Cell Biol. 5, 133–147 10.1038/nrm1313 15040446

[15] Prakash B., Praefcke G.J., Renault L., Wittinghofer A. and Herrmann C. (2000) Structure of human guanylate-binding protein 1 representing a unique class of GTP-binding proteins. Nature 403, 567–571 10.1038/35000617 10676968

[16] Liang K., Zhou G., Zhang Q., Li J. and Zhang C. (2014) Expression of hippo pathway in colorectal cancer. Saudi J. Gastroenterol. 20, 188–194 10.4103/1319-3767.133025 24976283 PMC4067916

[17] Santucci M., Vignudelli T., Ferrari S., Mor M., Scalvini L., Bolognesi M.L. et al. (2015) The hippo pathway and YAP/TAZ-TEAD protein-protein interaction as targets for regenerative medicine and cancer treatment. J. Med. Chem. 58, 4857–4873 10.1021/jm501615v 25719868

[18] Steinhardt A.A., Gayyed M.F., Klein A.P., Dong J., Maitra A., Pan D. et al. (2008) Expression of Yes-associated protein in common solid tumors. Hum. Pathol. 39, 1582–1589 10.1016/j.humpath.2008.04.012 18703216 PMC2720436

[19] Wang L., Shi S., Guo Z., Zhang X., Han S., Yang A. et al. (2013) Overexpression of YAP and TAZ is an independent predictor of prognosis in colorectal cancer and related to the proliferation and metastasis of colon cancer cells. PLoS ONE 8, e65539 10.1371/journal.pone.0065539 23762387 PMC3677905

[20] Hucl T., Brody J.R., Gallmeier E., Iacobuzio-Donahue C.A., Farrance I.K. and Kern S.E. (2007) High cancer-specific expression of mesothelin (MSLN) is attributable to an upstream enhancer containing a transcription enhancer factor dependent MCAT motif. Cancer Res. 67, 9055–9065 10.1158/0008-5472.CAN-07-0474 17909009

[21] Knight J.F., Shepherd C.J., Rizzo S., Brewer D., Jhavar S., Dodson A.R. et al. (2008) TEAD1 and c-Cbl are novel prostate basal cell markers that correlate with poor clinical outcome in prostate cancer. Br. J. Cancer 99, 1849–1858 10.1038/sj.bjc.6604774 19002168 PMC2600693

[22] Yuan H., Liu H., Liu Z., Zhu D., Amos C.I., Fang S. et al. (2015) Genetic variants in Hippo pathway genes YAP1, TEAD1 and TEAD4 are associated with melanoma-specific survival. Int. J. Cancer 137, 638–645 10.1002/ijc.29429 25628125 PMC4437894

[23] Stürzl M., Gaus D., Dirks W.G., Ganem D. and Jochmann R. (2013) Kaposi's sarcoma-derived cell line SLK is not of endothelial origin, but is a contaminant from a known renal carcinoma cell line. Int. J. Cancer 132, 1954–1958 10.1002/ijc.27849 22987579

[24] Chen C.A. and Okayama H. (1988) Calcium phosphate-mediated gene transfer: a highly efficient transfection system for stably transforming cells with plasmid DNA. BioTechniques 6, 632–638 PMID:3273409

[25] Held C., Nattkemper T., Palmisano R. and Wittenberg T. (2013) Approaches to automatic parameter fitting in a microscopy image segmentation pipeline: an exploratory parameter space analysis. J. Pathol. Inform. 4, S5 10.4103/2153-3539.109831 23766941 PMC3678745

[26] Malpica N., de Solorzano C.O., Vaquero J.J., Santos A., Vallcorba I., Garcia-Sagredo J.M. et al. (1997) Applying watershed algorithms to the segmentation of clustered nuclei. Cytometry 28, 289–297 10.1002/(SICI)1097-0320(19970801)28:4<289::AID-CYTO3>3.0.CO;2-7 9266748

[27] Unterer B., Becker C.M. and Villmann C. (2012) The importance of TM3-4 loop subdomains for functional reconstitution of glycine receptors by independent domains. J. Biol. Chem. 287, 39205–39215 10.1074/jbc.M112.376053 22995908 PMC3493960

[28] Ostler N., Britzen-Laurent N., Liebl A., Naschberger E., Lochnit G., Ostler M. et al. (2014) Gamma interferon-induced guanylate binding protein 1 is a novel actin cytoskeleton remodeling factor. Mol. Cell. Biol. 34, 196–209 10.1128/MCB.00664-13 24190970 PMC3911287

[29] Basu S., Totty N.F., Irwin M.S., Sudol M. and Downward J. (2003) Akt phosphorylates the Yes-associated protein, YAP, to induce interaction with 14-3-3 and attenuation of p73-mediated apoptosis. Mol. Cell 11, 11–23 10.1016/S1097-2765(02)00776-1 12535517

[30] Behrens J., Jerchow B.A., Wurtele M., Grimm J., Asbrand C., Wirtz R. et al. (1998) Functional interaction of an axin homolog, conductin, with beta-catenin, APC, and GSK3beta. Science 280, 596–599 10.1126/science.280.5363.596 9554852

[31] Tovchigrechko A. and Vakser I.A. (2006) GRAMM-X public web server for protein-protein docking. Nucleic Acids Res. 34, W310–W314 10.1093/nar/gkl206 16845016 PMC1538913

[32] Anbanandam A., Albarado D.C., Nguyen C.T., Halder G., Gao X. and Veeraraghavan S. (2006) Insights into transcription enhancer factor 1 (TEF-1) activity from the solution structure of the TEA domain. Proc. Natl Acad. Sci. U.S.A. 103, 17225–17230 10.1073/pnas.0607171103 17085591 PMC1859914

[33] Sayle R.A. and Milner-White E.J. (1995) RASMOL: biomolecular graphics for all. Trends Biochem. Sci. 20, 374 10.1016/S0968-0004(00)89080-5 7482707

[34] Fields S. and Song O. (1989) A novel genetic system to detect protein-protein interactions. Nature 340, 245–246 10.1038/340245a0 2547163

[35] Hollenberg S.M., Sternglanz R., Cheng P.F. and Weintraub H. (1995) Identification of a new family of tissue-specific basic helix-loop-helix proteins with a two-hybrid system. Mol. Cell. Biol. 15, 3813–3822 10.1128/MCB.15.7.3813 7791788 PMC230620

[36] Vojtek A.B., Hollenberg S.M. and Cooper J.A. (1993) Mammalian Ras interacts directly with the serine/threonine kinase Raf. Cell 74, 205–214 10.1016/0092-8674(93)90307-C 8334704

[37] Katoh M., Igarashi M., Fukuda H. and Nakagama H. (2013) Cancer genetics and genomics of human FOX family genes. Cancer Lett. 328, 198–206 10.1016/j.canlet.2012.09.017 23022474

[38] Mizuno T., Murakami H., Fujii M., Ishiguro F., Tanaka I., Kondo Y. et al. (2012) YAP induces malignant mesothelioma cell proliferation by upregulating transcription of cell cycle-promoting genes. Oncogene 31, 5117–5122 10.1038/onc.2012.5 22286761

[39] Zhou Y., Huang T., Cheng A.S., Yu J., Kang W. and To K.F. (2016) The TEAD family and its oncogenic role in promoting tumorigenesis. Int. J. Mol. Sci. 17, E138 10.3390/ijms17010138 26805820 PMC4730377

[40] Zhao B., Ye X., Yu J., Li L., Li W., Li S. et al. (2008) TEAD mediates YAP-dependent gene induction and growth control. Genes Dev. 22, 1962–1971 10.1101/gad.1664408 18579750 PMC2492741

[41] Yu M.H. and Zhang W. (2016) TEAD1 enhances proliferation via activating SP1 in colorectal cancer. Biomed. Pharmacother. 83, 496–501 10.1016/j.biopha.2016.06.058 27434865

[42] Kelleher F.C. and O'Sullivan H. (2016) FOXM1 in sarcoma: role in cell cycle, pluripotency genes and stem cell pathways. Oncotarget 7, 42792–42804 10.18632/oncotarget.8669 27074562 PMC5173172

[43] Laug R., Fehrholz M., Schutze N., Kramer B.W., Krump-Konvalinkova V., Speer C.P. et al. (2012) IFN-γ and TNF-α synergize to inhibit CTGF expression in human lung endothelial cells. PLoS ONE 7, e45430 10.1371/journal.pone.0045430 23029004 PMC3447888

[44] Vita M. and Henriksson M. (2006) The Myc oncoprotein as a therapeutic target for human cancer. Semin. Cancer Biol. 16, 318–330 10.1016/j.semcancer.2006.07.015 16934487

[45] Capaldo C.T., Beeman N., Hilgarth R.S., Nava P., Louis N.A., Naschberger E. et al. (2012) IFN-γ and TNF-α-induced GBP-1 inhibits epithelial cell proliferation through suppression of β-catenin/TCF signaling. Mucosal. Immunol. 5, 681–690 10.1038/mi.2012.41 22692453 PMC3481006

[46] Sakoda H., Gotoh Y., Katagiri H., Kurokawa M., Ono H., Onishi Y. et al. (2003) Differing roles of Akt and serum- and glucocorticoid-regulated kinase in glucose metabolism, DNA synthesis, and oncogenic activity. J. Biol. Chem. 278, 25802–25807 10.1074/jbc.M301127200 12734207

[47] Yoo G., Kim T., Chung C., Hwang D.S. and Lim D.S. (2017) The novel YAP target gene, SGK1, upregulates TAZ activity by blocking GSK3β-mediated TAZ destabilization. Biochem. Biophys. Res. Commun. 490, 650–656 10.1016/j.bbrc.2017.06.092 28634071

[48] Jiao S., Wang H., Shi Z., Dong A., Zhang W., Song X. et al. (2014) A peptide mimicking VGLL4 function acts as a YAP antagonist therapy against gastric cancer. Cancer Cell 25, 166–180 10.1016/j.ccr.2014.01.010 24525233

[49] Itsui Y., Sakamoto N., Kakinuma S., Nakagawa M., Sekine-Osajima Y., Tasaka-Fujita M. et al. (2009) Antiviral effects of the interferon-induced protein guanylate binding protein 1 and its interaction with the hepatitis C virus NS5B protein. Hepatology 50, 1727–1737 10.1002/hep.23195 19821486

[50] Zhu Z., Shi Z., Yan W., Wei J., Shao D., Deng X. et al. (2013) Nonstructural protein 1 of influenza A virus interacts with human guanylate-binding protein 1 to antagonize antiviral activity. PLoS ONE 8, e55920 10.1371/journal.pone.0055920 23405236 PMC3566120

[51] Zou Z., Meng Z., Ma C., Liang D., Sun R. and Lan K. (2017) Guanylate-binding protein 1 inhibits nuclear delivery of Kaposi's sarcoma-associated herpesvirus virions by disrupting formation of actin filament. J. Virol. 91, e00632-17 10.1128/JVI.00632-17 28592529 PMC5533911

[52] Persico M., Petrella L., Orteca N., Di Dato A., Mariani M., Andreoli M. et al. (2015) GTP is an allosteric modulator of the interaction between the guanylate-binding protein 1 and the prosurvival kinase PIM1. Eur. J. Med. Chem. 91, 132–144 10.1016/j.ejmech.2014.07.093 25081641

[53] Forster F., Paster W., Supper V., Schatzlmaier P., Sunzenauer S., Ostler N. et al. (2014) Guanylate binding protein 1-mediated interaction of T cell antigen receptor signaling with the cytoskeleton. J. Immunol. 192, 771–781 10.4049/jimmunol.1300377 24337748

[54] Lee J.D., Jung H. and Min S.H. (2016) Identification of proteins suppressing the functions of oncogenic phosphatase of regenerating liver 1 and 3. Exp. Ther. Med. 12, 2974–2982 10.3892/etm.2016.3722 27882103 PMC5103732

