# Supplementary material for: IFN-γ-response mediator GBP-1 represses human cell proliferation by inhibiting the Hippo signaling transcription factor TEAD
Source: Biochem J. 2018 Sep 25;475(18):2955–67. doi: 10.1042/BCJ20180123 (PMC6156764; doi:10.1042/BCJ20180123)
Supplement: Supplementary Figures and Table [file BCJ-475-2955-s1.pdf]

Supplementary Material

IFN-γ-response mediator GBP-1 represses human cell proliferation by inhibiting the Hippo signaling transcription factor TEAD

Supplementary Figure 1

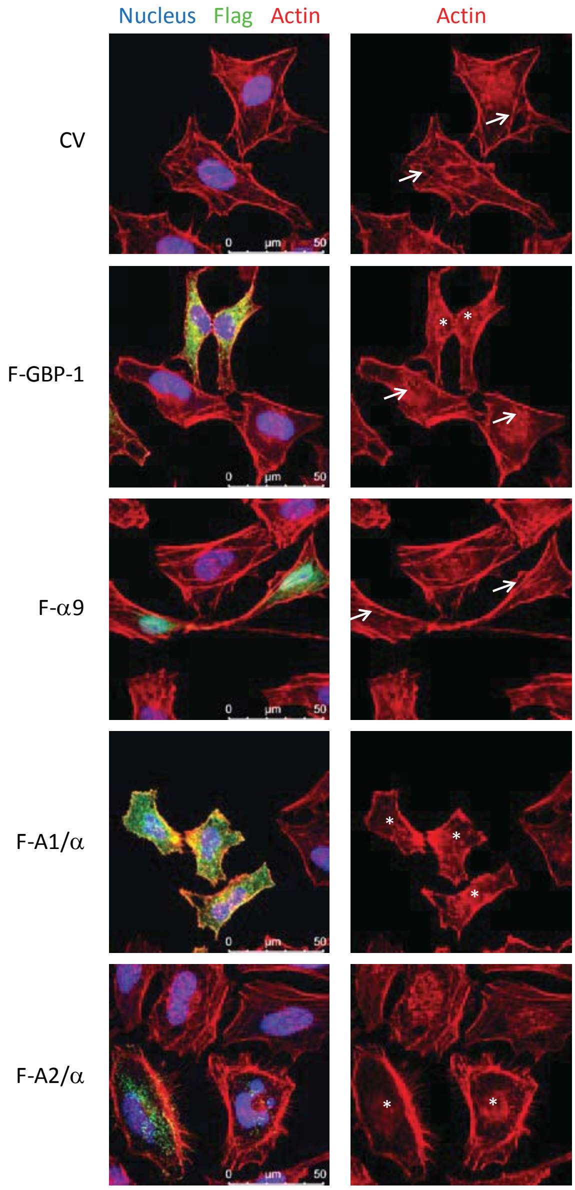

Supplementary Figure 1. The α9-helix of GBP-1 does not influence the actin cytoskeleton architecture. HeLa cells were transiently transfected with the indicated Flag-tagged expression plasmids or the plain vector (CV) as control. Transfected cells were detected using an anti-Flag antibody (Flag, green). The actin cytoskeleton was visualized using Phalloidin-AlexaFluor546 (red) and nuclei were counterstained with Draq5 (blue). Scale bars represent 50 µm. Intact actin fibers are indicated by arrows and disturbed actin cytoskeleton by asterisks.

Supplementary Figure 2

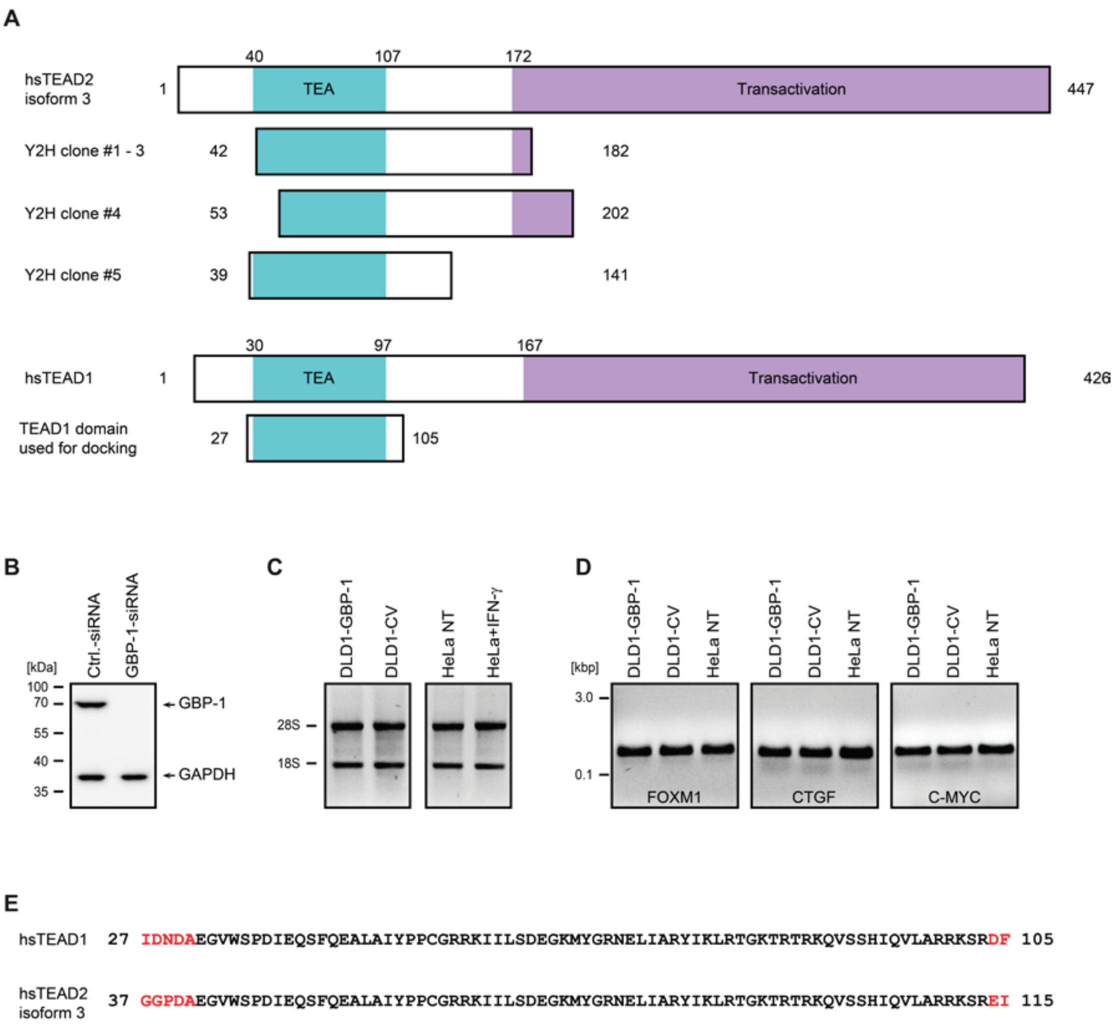

Su**pplementary Figure 2**. (**A**) Domain architecture of TEAD fragments that interact with the GBP-1-α9-helix. Five partly overlapping TEAD clones identified in the Y2H analysis are shown. Amino acid numbers correspond to human TEAD2 isoform 3 (NP_003589.1), consisting of an N-terminal TEA domain that binds to DNA and a C-terminal transactivation domain that binds co-activators. Below, hsTEAD1 and its domain that was used for molecular docking analysis is depicted. (**B**) GBP-1 expression is inhibited by siRNA. GBP-1 expression in cell lysates used for the luciferase assay was analyzed by western blot. GBP-1 expression was inhibited in cells treated with a specific siRNA (siGBP) but not with the control siRNA (siCtrl.). GAPDH staining confirmed the amount of protein analyzed was equal. (**C**) RNA integrity. RNA was isolated from DLD1-cells stably expressing GBP-1 (DLD1-GBP-1) or transfected with the empty vector (DLD1-CV) and from HeLa cells either unstimulated or stimulated with IFN-γ and subjected to agarose gel electrophoresis. RNA was intact as indicated by 18 S and 28 S rRNA. (**D**) Primers were specific for TEAD target genes. Endpoint PCR products (after 40 cycles) showed for each of the different genes one specific band of the correct size in agarose-gel electrophoresis. (**E**) TEAD sequence alignment. TEAD1 (NP_068780.2) residues 27-105 used in molecular docking analyses correspond to hsTEAD2 isoform 3 residues 37-115. Not identical residues are marked in red.

Supplementary Table 1. Similarity Search

Protein	Blast	PattInProt
GBP-1	VDHLFQK	VDHLFQK
GBP-3	VDHLFQK	VDHLFQK
serine/threonine-protein phosphatase 6 regulatory subunit 2	VTHLFQK	VTHLFQK
ras-related protein Rab-24	VDELFQK	VDELFQK
potassium voltage-gated channel, subfamily H (eag-related), member 5	VDHPVRKLFQK	
heart muscle inducible nitric oxide synthase	-DHLYQK	
sprouty-related, EVH1 domain-containing protein 1	-DHLFQ-	
teneurin-2	--HLFQK	
active breakpoint cluster region-related protein isoform f	--HLFQK	
OCRL-1 gene	VDHLF--	

Transcriptome-wide search of the first seven α9 residues (376-382) was performed by Blast and PattInProt search. For the Blast search, all results are given. For the PattInProt search, only results with a maximum of one mismatch are listed because there are more than 100 results when the setting is changed to two mismatches. Changed residues are depicted in red.

Supplementary material and methods

Immunofluorescence analysis of the actin cytoskeleton

For immunofluorescence analysis of the actin cytoskeleton, HeLa cells were seeded in 4-well chamber slides (Nunc) at a cell density of 2.5×10^4 cells. Twenty-four hours after seeding, cells were transfected with 1 μg of plasmid per well using the calcium phosphate method. Cells were fixed with 4% buffered paraformaldehyde and permeabilized with 0.1% Triton X100 (both from Sigma-Aldrich) 48 h post-transfection. Transfected cells were detected using a polyclonal rabbit anti-Flag-tag antibody (1:500, Thermo Fisher Scientific, Rockford, IL USA). AlexaFluor 488-conjugated goat anti-rabbit IgG (1:500) was used as secondary antibody (Molecular Probes/Invitrogen). Actin was stained using fluorescently labeled phalloidin (Alexa Fluor® 546 Phalloidin, 1:70, Invitrogen/Molecular Probes) and nuclei were counterstained with Draq5 (1:800, Cell Signaling, Danvers, MA, USA). The coverslips were mounted with fluorescence mounting medium (Dako). Fluorescence was visualized using a confocal microscope, TCS SPE (Leica Microsystems, Wetzlar, Germany) using a 63x magnification. All images presented are single sections in the z-plane (1 airy unit).
